# The Obstetrical Journal of Great Britain and Ireland

**Published:** 1873-05-15

**Authors:** 


*The Obstetrical Journal of Great Britain and Ireland*, including Midwifery and the Diseases of Women and Children. Edited by James H. Aveling, M.D., and Alfred Wiltshers, M.D.; with an American Supplement, edited by Wm. F. Jenks, M.D. Philadelphia: Henry C. Lea.

The first issue of this new journal, the number for April, 1873, is now before us. It is to be issued in monthly numbers of 72 pages each, with an American Supplement of 16 pp. The subscription price is $5.00 per annum. The list of regular contributors includes such well known names as Dr. L. Athill, Dr. J. H. Bennett, Dr. Thos. Chambers, Dr. F. Churchill, Dr. Alfred Medows, and many others

The addition of an American Supplement will undoubtedly increase its sphere of usefulness in this country. The Supplement is intended to contain short original communications, or clinical lectures, and a thorough abstract of the most important and interesting articles relating to obstetrics and kindred branches, selected from the American periodical publications.

